# Antimicrobial Activity of *Psidium guajava* Aqueous Extract against Sensitive and Resistant Bacterial Strains

**DOI:** 10.3390/microorganisms11071784

**Published:** 2023-07-10

**Authors:** Geraldo Augusto Pereira, Douglas Siqueira de Almeida Chaves, Taynara Monsores e Silva, Raissa Emidio de Araújo Motta, Adriana Barbosa Rocha da Silva, Thereza Cristina da Costa Patricio, Anna Julia Bessa Fernandes, Shana de Mattos de Oliveira Coelho, Marcin Ożarowski, Yara Peluso Cid, Tomasz M. Karpiński

**Affiliations:** 1 Pos Graduation Program of Veterinary Science, Veterinary Institute, Federal Rural University of Rio de Janeiro, BR 465, km 7, Seropédica 23897-000, RJ, Brazil; 2 Pharmaceutical Science Department, Health and Biological Science Institute, Federal Rural University of Rio de Janeiro, BR 465, km 7, Seropédica 23897-000, RJ, Brazil; 3 Veterinary Microbiology and Immunology Department, Veterinary Institute, Federal Rural University of Rio de Janeiro, BR 465, km 7, Seropédica 23897-000, RJ, Brazil; 4 Department of Biotechnology, Institute of Natural Fibres and Medicinal Plants, Wojska Polskiego 71b, 60-630 Poznań, Poland; 5 Chair and Department of Medical Microbiology, Poznań University of Medical Sciences, Rokietnicka 10, 60-806 Poznań, Poland

**Keywords:** phytopharmaceuticals, *Psidium guajava*, antimicrobial resistance, phenolics, antioxidant

## Abstract

The inappropriate use of antimicrobials, along with environmental conditions, can lead to the emergence of resistant microorganisms. The use of phytopharmaceuticals and herbal medicines has a positive impact and represents a promising alternative. *Psidium guajava* extracts have been widely reported to have antimicrobial potential; however, studies reporting their activity against resistant bacterial strains are scarce. Because of the emerging resistance, the aim of this study was to analyze the antimicrobial capacity of the aqueous extract of guava leaves against wild-type and resistant bacterial strains. The aqueous extract obtained from the leaves of *P. guajava* was evaluated by HPLC for the content of total phenolics and tannins, antioxidant activity, and chemical composition. The antimicrobial activity of the extracts was analyzed by the disk diffusion and broth microdilution methods. The results of the chemical analysis of the extracts showed total phenolics content of 17.02 ± 6.87 mg/g of dry extract, total tannin content of 14.09 ± 1.20 mg of tannic acid equivalents/g of dry extract, and moderate antioxidant capacity with an EC_50_ value of 140 µg/mL. Flavonoids are the major compounds (rutin, hesperidin, and quercetin), followed by phenolic acids. Disk diffusion test results showed the presence of inhibition halos for Gram-positive bacteria (*Staphylococcus aureus,* sensitive and resistant; *Staphylococcus pseudintermedius,* sensitive and resistant; and *Streptococcus* spp., beta-hemolytic), while for Gram-negative bacteria (*Escherichia coli*, sensitive and resistant), there was no inhibition in the tested concentration range. The Minimal Inhibitory Concentration was 6.8 mg/mL for all Gram-positive strains evaluated. The present study demonstrated the antimicrobial activity of the aqueous extract of *P. guajava* against sensitive and resistant Gram-positive bacteria. The better antimicrobial activity found in the present study compared with previously reported activity should be highlighted and may be related to the higher concentration of total phenolics present in the tested extract. Moreover, the content of tannins found suggests a species with high quality that produces tannins. These new findings suggest an innovative profile regarding therapeutic resources that can be adopted to combat resistant microbial strains.

## 1. Introduction

Antimicrobial resistance can be described as the ability of a microorganism to resist the action of antimicrobials, which regularly occurs through continuous exposure to them. The level of resistance of a mutant strain can vary widely depending on the mechanism of resistance resulting in its evolution, either by spreading between similar or dissimilar strains [1]. The emergence and spread of antimicrobial-resistant bacteria have been classified by the World Health Organization (WHO), the United States Center for Disease Control and Prevention (CDC), and the European Center for Disease Prevention and Control (ECDC) not only as an emerging global disease but as one of the three most significant threats to public health in the 21st century [2]. The Review on Antimicrobial Resistance (2016) [3] report warned of the likelihood of a catastrophic increase in global rates of antimicrobial resistance (AMR), raising the 700,000 annual deaths attributable to infections by resistant pathogens to an alarming 10 million cases in 2050, with significant public spending in the order of USD 100 trillion.

The improper use of antimicrobials stimulated the emergence of genetic modifications that contributed to circumventing the mechanism of action of drugs. Therefore, the expansion of resistant strains results in damage to public health as it leads to infectious conditions that require difficult treatment [1]. The use of phytopharmaceuticals and herbal medicines has a positive impact on therapy, representing a promising alternative since many microorganisms have developed resistance to synthetic drugs [4,5].

*P. guajava*, popularly known as guava [6], is widely cultivated in Brazil. In addition to food, its various extracts have traditionally been used in Brazil for medicinal purposes. The origin of the guava tree is in tropical and subtropical areas of the Americas, with a prevalence in dry climates. Still, it expands naturally throughout Tropical America, between southern Mexico and northern South America. It is considered one of the tropical and subtropical fruits with high value and importance due to its natural sources of vitamins and minerals.

The chemical composition of *P. guajava* includes tannins such as guavins A-D and flavonoids [7,8], and its extracts have already been widely reported to have antimicrobial potential. Many studies have described the antimicrobial activity of different extracts from the leaves of *P. guajava*, such as methanolic [9,10,11,12,13,14,15,16], ethanolic [9,16,17,18,19,20], and aqueous [9,11,12,13,16,19,20,21,22,23], including the activity of methanolic extracts against methicillin-resistant *Staphylococcus aureus* (MRSA) strains [18]. The antimicrobial activity of the root [12] and fruit extracts [24] of *P. guajava* has also been reported.

Because of the emerging resistance of microorganisms, the aim of this study was to analyze the antimicrobial capacity of the aqueous extract of guava leaves against wild-type and resistant strains.

## 2. Materials and Methods

### 2.1. Chemicals and Reagents

Solvents for extraction and preliminary analysis such as methanol, Folin-Ciocalteu, DPPH (2,2-Diphenyl-1-picrylhydrazyl) (Sigma, Brazil), sodium carbonate, gallic acid, tannic acid, and ferric chloride were obtained from Sigma-Aldrich (Brazil). Ultrapure water was obtained from MilliQ equipment from Merck, São Paulo, Brazil. Culture medium (yeast extract, glucose, peptone, and agar) were acquired from Difco, IL, USA, and hydrogen peroxide and dimethylsulfoxide (DMSO) from Sigma-Aldrich, Brazil.

### 2.2. Plant Material, Extraction, and High Performance Liquid Chromatography

Leaves of *P. guajava* L. (Myrtaceae) were obtained from the Federal Rural University of Rio de Janeiro in Brazil (UFRRJ)—GPS 22°46′3″ S and 43°41′36″ W. A voucher specimen classified by Dr. Marcelo de Souza (RBRv90000025) is deposited in the Botanical Garden of the Botany Department of UFRRJ. The plant material was dried at 45 °C for 72 h in an oven with controlled air circulation. The dried guajava leaves were crushed using a knife mill and then submitted to extraction by decoction in water at 80 °C at 10% *w*/*v* for 15 min. After the material was filtered, concentrated, frozen, and lyophilized to obtain the final yield.

Identification of secondary metabolites present in the extracts of leaves from guajava was performed on a Shimadzu liquid chromatograph LC-20AT with a diode-array wavelength SPD-M20A detector using a Merck reverse-phase column C-18 (5 μm, 150 mm, 4 mm). The mobile phase consisted of water adjusted to pH 3.0 with 0.1% formic acid (eluent A) and methanol (eluent B). The samples were run for 45 min at 1 mL/min, and absorbance was monitored between 200 and 600 nm. The gradient used in chromatography analysis was 0 min = 10%B, 5 min = 10%B, 10 min = 30%B, 15 min = 30%B, 20 min = 70%B, 27 min = 90%B, 35 min = 90%B, 37 min = 10%B, and 45 min = 10%B. *Psidium guajava* extract (2.5 mg) was dissolved in deionized water (2 mL), ultrasonicated (30 min), and filtered on a Millipore filter. An amount of 20 μL was injected for analysis. Tannic acid (0.5 mg/mL), gallic acid (0.5 mg/mL), protocatechuic acid (0.5 mg/mL), chlorogenic acid (0.5 mg/mL), syringic acid (0.5 mg/mL), ellagic acid (0.5 mg/mL), rosmarinic acid (0.5 mg/mL), vitexin (0.5 mg/mL), quercetin (0.5 mg/mL), and rutin (0.5 mg/mL) were used as standards. Phenolics and flavonoids were quantified by the area of the extract.

### 2.3. Total Phenolic and Tannin Content

The total phenolic content was determined by the Folin-Ciocalteu (FC) method using a standard curve of an alcoholic solution of gallic acid at five different concentrations (0, 1, 3, 5, 7, and 9 µg/mL). To these dilutions, 5.0 mL of water and 2.5 mL of diluted Folin-Ciocalteu reagent (1:10 in distilled water) were added. From a methanolic solution of the extract (1 mg/mL), a 0.5 mL aliquot was placed in an amber flask with a cap, and 5.0 mL of water and 2.5 mL of FC solution (10% *v*/*v*) were added, stirred quickly, and left to stand for 5 min. A 2.0 mL aliquot of a 4% sodium carbonate solution was added, and the mixture was allowed to stand for 2 h, after which the optical density was measured at 765 nm against a blank. The total phenolic contents were calculated on the basis of the calibration curve of gallic acid and expressed as gallic acid equivalents (GAE) in milligrams per g of dry extract [25].

The total tannin content in the aqueous guava leaf extract was determined using the aluminum chloride colorimetric method, adapted from Quettier-Deleu et al. [26]. For this, aliquots of 0.5 mL, in triplicate, of each sample of the hydroalcoholic extract were added to an equal volume of a methanolic solution of 5% aluminum chloride (AlCl_3_). After standing for 15 min, the absorbance was read at 420 nm. Total tannin content was determined using a standard tannic acid curve at concentrations of 0, 5, 10, 20, 30, 40, and 50 µg/mL. Samples were independently analyzed in triplicate, and the total tannin content was expressed as mg tannic acid (TAE) equivalents per g of dry extract.

### 2.4. Antioxidant Activity

The antioxidant activity of the extract was evaluated by the DPPH free radical assay, which involves the measurement of the decrease in absorbance of the extract (using a UV spectrophotometer). The stabilization of free radicals is seen by the change in color from dark violet to light violet [27]. Aqueous solutions were composed of 50% methanol and 70% acetone, 0.06 mM methanolic solution, and methanolic dilutions of the extract (sample) (1, 2, 5, 7, and 10 μg/mL). The reaction mixture was produced by adding 0.1 mL of sample to 3.9 mL of 0.06 mM DPPH solution. The absorbance reading was measured at 515 nm in pentaplicate. The DPPH calibration curve (1, 2, 3, 4, and 5 μM) used methanol as a blank to create the first linear equation. At the same wavelength, another reading was taken after 55 min of reaction, and the EC_50_ curve (sample and DPPH), in triplicate, was plotted to create the second linear equation. The elimination activity value, EC_50_, expresses the amount of extract needed to decrease the absorbance of DPPH by 50%, which was determined graphically by plotting the linear regression of the absorbance against the extract concentration. This experiment followed the method described by Rufino et al. [27] and De Menezes Epifanio et al. [25].

### 2.5. Antimicrobial Activity of the Extract

The antimicrobial activity of the extract was analyzed by the disk diffusion and broth microdilution methods to determine the initial concentration of extract capable of enlarging the inhibition halo and to determine the minimum inhibitory concentration (MIC) and minimum bactericidal concentration (MBC), respectively. For the antimicrobial evaluation, dilutions of the extract (in water) were tested against the strains *Escherichia coli* ESBL (CMY-2), *Escherichia coli* (ATCC 25922), *Staphylococcus aureus* MRSA (ATCC 43300), *Staphylococcus aureus* (ATCC 23923), *Staphylococcus pseudintermedius* (B19), *Staphylococcus pseudintermedius* (B20), and *Streptococcus* spp. beta-hemolytic, described in Table 1.

#### 2.5.1. Disk Diffusion Test

The strains were incubated for 24 h on brain heart infusion (BHI) agar at 35 °C. Filter paper discs (0.38 cm^2^) impregnated with different concentrations of the extract (2.3, 4.6, 6.8, 9.1, and 11.4 mg/mL) were used, placed on Mueller-Hinton (MH) agar plates inoculated with the strains previously adjusted to 0.5 on the McFarland scale, and incubated for 24 h [28]. The tests were carried out in triplicate, and the impregnation volume was 6 μL, using water and 10% methanol as blanks.

#### 2.5.2. Broth Microdilution Test

The broth microdilution test was carried out using a 96-well microtiter plate to evaluate the minimum inhibitory concentration (MIC). The strains were previously inoculated in MH broth at 35 °C and adjusted to 0.5 on the McFarland scale. Concentrations of *P. guajava* aqueous extract were set based on the results of the disk diffusion test. Imipenem was used as a negative control. For positive control, only strains without extract were used, and the blank was broth with extract. UV spectroscopy was used at λ 655 nm [29] to measure the turbidity, and the MIC was determined by the concentration of the extract of the sample that did not show turbidity when compared to the blank.

#### 2.5.3. Minimum Bactericidal Concentration Determination

The minimum bactericidal concentration (MBC) was evaluated with aliquots from the sample wells (strain and extract), where no turbidity was observed, inoculated in BHI culture medium at 36 °C, and read after 24 h. The MBC was defined as the lowest concentration of the extract capable of preventing the growth (meaning death) of the inoculum [28].

### 2.6. Statistical Analysis

In the statistical analysis, the averages of the inhibition halos and turbidity were compared. Initially, the data were assessed for normal distribution using the Shapiro-Wilk test. The data with a normal distribution (parametric) were submitted to analysis of variance (ANOVA) and the Tukey test. The data that did not present a normal distribution (nonparametric) were evaluated using the Kruskal–Wallis test. The level of significance considered in all tests was 95% (*p* ≤ 0.05). Statistical analyses were performed using the GraphPad Prism 7 statistical program (GraphPad Software Inc., San Diego, CA, USA).

## 3. Results and Discussion

### 3.1. Chemical Analysis of Plant Extract

**Figure 1:**
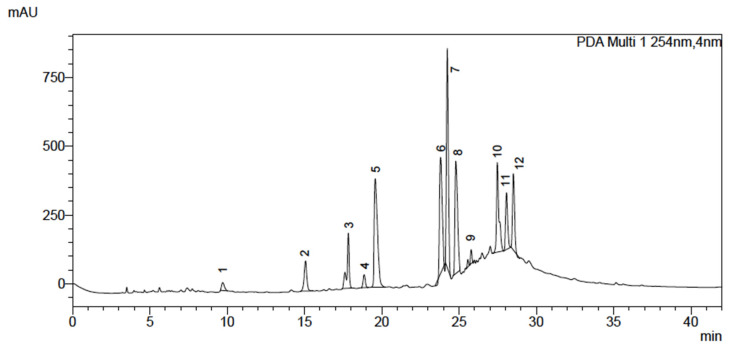
HPLC analyzes of *Psidium guajava* crude extract using co-injection of standards. (1—tannic acid, 2—gallic acid, 3—protocatechuic acid, 4—rutin, 5—hesperidin, 6—quercetin, 7—cinnamic derivative, 8—not identified, 9—syringic acid, 10—cinnamic derivative, 11—ellagic acid, 12—rosmarinic acid).

HPLC analysis led to the identification of 12 peaks with different λ_max_. benzoic acids, phenolic acids, and flavonoids. The chemical analyses are shown in Figure 1 and Table 2.

The compound 1 (R_t_ = 9.711 min) was identified as tannic acid, followed by gallic acid (2, R_t_ = 15.076 min). The compound 3 (R_t_ = 17.838 min) shows λ_max_ 242 and 294 nm, corresponding to protocatechuic acid. Rutin, quercetin, hesperidin, and cinnamic derivatives were identified as major compounds (4–7), and compound 8 was not identified. Other compounds such as syringic, ellagic, and rosmarinic acids can be found in the guava extract.

*P. guajava* is a species rich in phenolic chemical compounds in its leaves, such as flavonoids (+)-psiflavanone A, (−)-psiflavanone A, (+)-psiflavanone B, (−)-psiflavanone B [8], The yield of the extract was 4.3% (*w*/*w*), similar to reports in the literature. The total phenolic content of the aqueous extract, quantified from the standard curve (y = 0.1401x + 0.0047; *R*^2^ = 0.9971), was 17.02 ± 6.87 mg/g of dry extract. The total tannin content of the aqueous extract was quantified using a standard curve equation: y = 0.0018x + 0.0328; R^2^ = 0.9741, affording 14.09 ± 1.20 mg of tannic acid equivalents/g of dry extract. A comparison of our results with those reported in the literature indicated significant differences in the guava leaf extracts’ phenolic compounds. However, the content of tannins found in our study suggests a species with high quality that produces tannins.

Nantitanon et al. [30] investigated the influence of certain factors on the yield, antioxidant activity (AA), and total phenolic content (TPC) of guava leaf extract, as well as the effects of pretreatment of leaf samples prior to extraction, the extraction method, and the leaf age. Folin–Ciocalteu was used to determine the TPC, and the values reported ranged from 80.28 mg ± 1.58 to 136.02 mg ± 5.55 EAG/g of extract from the different extraction methods (maceration with/without stirring, ultrasonication, and Soxhlet extraction). Haida et al. [31] found phenolic contents ranging from 158.29 to 165.07 EAG mg/g dry extract of white guava and from 160.61 to 175.10 EAG mg/g for red guava. Camarena-Tello et al. [32] reported the total phenolic compounds of *P. guajava* in different solvents. The acetone fraction had the greatest quantity of phenolic compounds of both varieties, followed by the aqueous fraction, and finally the chloroform fraction (71.69 ± 3.69–374.63 ± 29.92 mg GAE/g extract).

Some authors have reported that the pretreatment process of the guava leaves before extraction and the extraction method are important factors that affect the amount of active principles and antioxidant activity of the extracts. The maturity stage of the guava leaves and the extraction solvent are other important factors that result in different phenolic contents.

The genus *Psidium* belongs to the Myrtaceae family and comprises important botanical species, especially the guava tree (*Psidium guajava* L.). The health benefits of the phenolic composition of guava fruits and leaves have been studied due to their chemical composition and pharmacological properties, such as antifungal and antimicrobial activities.

From the absorbances obtained from the different dilutions of *P. guajava* extract, it was possible to calculate the total antioxidant activity (EC_50_) as the absorbance equivalent to 50% of the DPPH concentration by the standard curves of DPPH (y = 0.1415x − 0.005). So, for the aqueous extract from the leaves of *P. guajava*, we obtained an EC_50_ of 140.0 µg/mL (y = −0.044x + 0.7214), considered to be the moderate antioxidant capacity of the extract, corroborating the results described by Iha et al. [24] (EC_50_ = 150.0 µg/mL) and Camarena-Tello et al. [32] (EC_50_ = 269.78 µg/mL).

### 3.2. Antimicrobial Extract Evaluation

**Figure 2:**
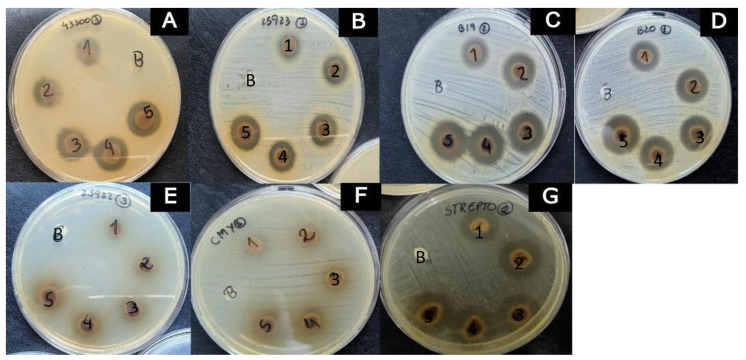
Inhibition halos presented by *Psidium guajava* aqueous extract in the concentration range of 2.3 to 11.4 mg/mL (1–5) and blank (**B**) against the different strains: (**A**) *Staphylococcus aureus* (resistant); (**B**) *Staphylococcus aureus* (wild-type); (**C**) *Staphylococcus pseudintermedius* (resistant); (**D**) *Staphylococcus pseudintermedius* (wild-type); (**E**) *Escherichia coli* (wild-type); (**F**) *Escherichia coli* (resistant); (**G**) *Streptococcus* beta-hemolytic (wild-type).

In the disk diffusion tests, inhibition halos were found for Gram-positive bacteria, while for Gram-negative bacteria (*Escherichia coli*, sensitive and resistant), there was no inhibition at the tested concentrations (Table 3 and Figure 2), corroborating the results reported by Araújo et al. [33] and Kidaha et al. [12], where no inhibition was found for *E. coli*. Gram-negative bacteria are generally more resistant to antimicrobials than Gram-positive bacteria because they have an additional outer membrane that can protect them from antimicrobial compounds [34].

At the highest concentration evaluated (11.4 mg/mL), we observed inhibition halo values of 18.0 ± 3.46 mm for resistant *S. aureus*, 16.0 ± 0 mm for sensitive *S. aureus*, 17.3 mm ± 1.15 for resistant *S. pseudintermedius*, 18 mm ± 0 for sensitive *S. pseudintermedius* and 21 mm ± 2.00 for sensitive *Streptococcus beta-hemolytic*. Inhibition halos were dose-dependent for the gram-positive bacteria tested. The same had already been reported by Bolzan et al. [17], who observed dose-dependence in the inhibition halos obtained against the ethanolic extract of *P. guajava* leaves in a clinical trial carried out with microorganisms from the canine oral microbiota.

The activity of the aqueous extract of *P. guajava* against *S. aureus* through the disk diffusion test has already been reported in previous studies, but the concentrations necessary to obtain the inhibition halo were higher, 50.0 mg/mL [16] and 62.5 mg/mL [33], compared to our results (11.40 mg/mL). The better antimicrobial activity found in the present study may be related to the higher concentration of total phenolics present in the tested extract than the values reported by Raj et al. [16] and Araújo et al. [33], since these compounds generally exhibit antibacterial activity.

**Figure 3:**
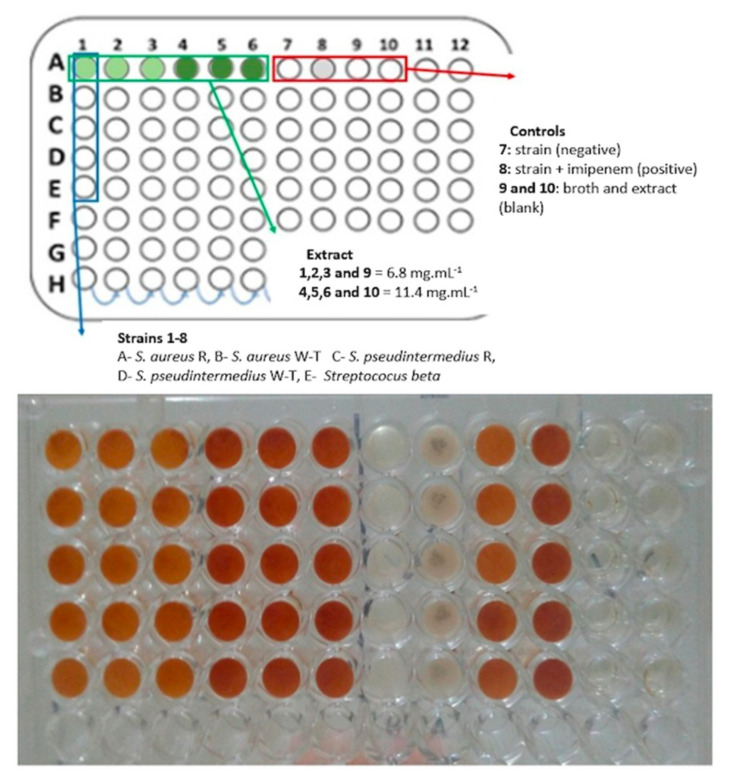
Minimum Inhibition Concentration determination: broth microdilution test of *Psidium guajava* aqueous extract in the concentrations of 6.8 mg/mL (1–3 and 9) and 11.4 mg/mL (4–6 and 10) against the different strains: (A) *Staphylococcus aureus* (resistant); (B) *Staphylococcus aureus* (wild-type); (C) *Staphylococcus pseudintermedius* (resistant); (D) *Staphylococcus pseudintermedius* (wild-type); (E) *Streptococcus* beta-hemolytic (wild-type). Negative control imipenem (8). F, G and H were not utilized in the experiment.

Based on the results obtained in the disk diffusion test, concentrations of 6.8 and 11.40 mg/mL, respectively, were selected for MIC and MBC evaluations. The broth microdilution assay demonstrated that microbial growth inhibition occurred in all strains at both concentrations tested (6.8 and 11.40 mg/mL) (Figure 3). Therefore, the MIC was 6.8 mg/mL for all strains, a value similar to that reported by Metwally et al. [35] (5.25 mg/mL) for *S. aureus* (sensitive), higher than that reported by Sanches et al. [20] (500 µg/mL) for *S. aureus* (sensitive), and lower than the values reported by Araújo et al. [33] (62.5 mg/mL) for *S. aureus* (sensitive and resistant) and Ratnakara et al. [13] (30–40 mg/mL) for *S. aureus* (sensitive).

**Figure 4:**
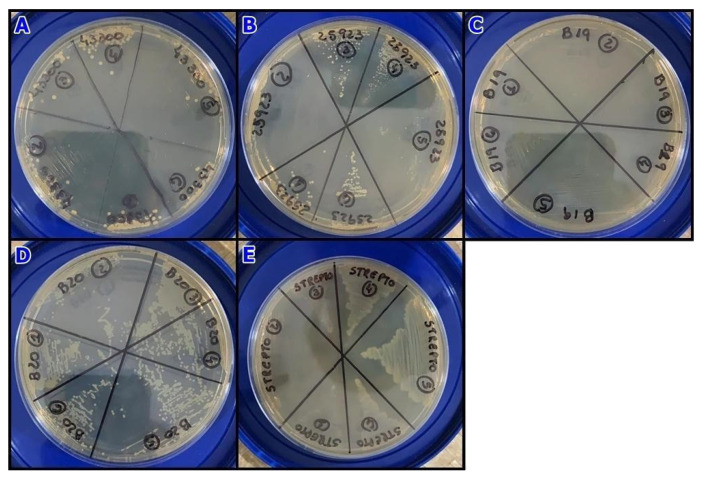
Minimum bactericidal concentration determination: aliquot-seeded BHI plates after broth microdilution test. (**A**) *Staphylococcus aureus* (resistant); (**B**) *Staphylococcus aureus* (wild-type); (**C**) *Staphylococcus pseudintermedius* (resistant); (**D**) *Staphylococcus pseudintermedius* (wild-type); (**E**) *Streptococcus* beta-hemolytic (wild-type).

The results of MBC determination indicated that for *S. pseudintermedius* (resistant), the interaction between extract and microorganism prevented growth at a concentration of 6.8 mg/mL. However, for the other strains, the MBC value was greater than 11.4 mg/mL (Figure 4).

Most previous works highlight the antimicrobial activity of the aqueous extract of *P. guajava* only against sensitive strains of Gram-positive bacteria, finding little or no effectiveness against resistant strains. Likewise, the large variation in inhibitory concentration between the studies suggests that it may be related to the proportion of total phenolic and tannins present in each extract, which in turn may be a consequence of the extraction method or the exposure of plant material to different environmental conditions, as reported by Raj et al. [16] and Biswas et al. [9]. Moorthy et al. [36] suggest that tannins would be primarily responsible for antimicrobial activity.

The influence of the extraction method, the part of the plant, and the extracting solvent on the antimicrobial activity was demonstrated in a study carried out by Sanches et al. [20]. The aqueous extract of leaves, roots, and stem bark of *P. guajava* showed different potencies against *S. aureus* (MICs = 500, 125, and 250 µg/mL, respectively) and practically inactivity against Gram-negative bacteria such as *E. coli* and *P. aeruginosa* (MICs > 1000 µg/mL). Ethanol: water extracts showed greater antimicrobial activity when compared to aqueous extracts.

In comparative studies of aqueous, ethanolic, and methanolic extracts, the methanolic extract had greater activity against gram-positive and gram-negative bacteria than the others, as reported by Biswas [9], Dhiman [10], Ratnakaran [13], and Nair [14].

Furthermore, for biological purposes, it is important to analyze the association between extracts from different plant species. The combination of extracts of *P. guajava* and *Cannabis sativa,* or *Trema orientalis*, was able to promote inhibition against MRSA. The same activity was observed when analyzing clinical samples of the same strain [18]. Another study demonstrated the synergy between aqueous extracts of *Psidium* sp., *Mongifera* sp., and *Mentha* sp. against *Streptococcus* sp. [23].

Likewise, it is also important to evaluate the potential activity of the different major chemical components as well as search for additive or synergistic activity from the combination of major components in the same plant extract [37]. The flavonoids luteolin, morin, naringin, rutin, and quercetin were effective in inhibiting the growth of gram-positive and gram-negative bacteria of different genera, including *S. aureus* and *E. coli* [38]. Previously, Amin et al. [39] indicated combined inhibitory activity against MRSA by the flavonoids morin, rutin, and quercetin. In addition, it demonstrated additive efficacy when these isolates were associated with existing commercial antibiotics.

It is important to highlight the significant evidence of the activity of the aqueous extract of *P. guajava* against resistant strains of zoonotic importance, such as *S. aureus* and *S. pseudintermedius*, observed by us, since there is a lack of studies reporting the activity of *P. guajava* extracts against resistant strains.

As previously described by Gelatti [40] and Hughes [1], certain strains have become less sensitive to different antimicrobials, thus indicating the importance of searching for new forms of treatment to overcome resistance mechanisms. More specifically, Donkor [41] and Jaradat [42] highlighted the problem of MRSA, with a high incidence of transmission associated with the oral mucosa, as also previously reported by Turner [43], who described the threat this scenario poses to public health. In addition to its extensive resistance to antibiotics, MRSA is a cause of serious concern due to the high prevalence of its infections and association with persistent outbreaks, which have serious economic implications [41].

Due to the problematic bacterial resistance in Brazil and around the world, a deeper study of the potential of plants and their metabolites as therapeutic alternatives capable of combating multiresistant microorganisms becomes significant and necessary. Mainly because it is a more inexpensive and accessible treatment, especially considering that Brazil is a country of outstanding biodiversity.

## 4. Conclusions

The present study demonstrates the antimicrobial activity of the aqueous extract of *P. guajava* leaves against sensitive and resistant Gram-positive bacteria. New findings suggest an innovative profile regarding therapeutic resources that can be adopted to combat resistant microbial strains. According to the estimate of the World Health Organization for 2050, it is important to continue the studies of this species and its compounds in the search for a new innovative antibacterial drug.

## Figures and Tables

**Table 1:** Description of the strains tested against the guava leaf extract.

Strains	Code	Resistance Pattern
*E. coli* (ESBL)	CMY-2	Betalactamic resistance (Penicillins and cephalosporins up to third generation)
*E.* *coli*	ATCC 25922	Wild-type *E. coli*
*S. aureus* (MRSA)	ATCC 43300	Methicillin resistant
*S.* *aureus*	ATCC 23923	Wild-Type *S. aureus*
*S.* *pseudintermedius*	B19	Resistant to Sulfametoxazole + trimetoprim
*S.* *pseudintermedius*	B20	No expressed resistance to any tested antimicrobials
*Streptococcus* spp. beta-hemolytic	-	No expressed resistance to any tested antimicrobials

Legend: ESBL—Extended Spectrum Betalactamase; MRSA—Methicillin resistant *Staphylococcus aureus*.

**Table 2:** Chemical composition identified from *Psidium guajava* extract.

Peak	Retention Time (R_t_)	Concentration (%)	λ_max_ (nm)	Name
1	9.711	1.079	278	Tannic acid
2	15.068	3.699	270	Gallic acid
3	17.838	1.316	242, 294	Protocatechuic acid
4	18.865	16.549	257, 358	Rutin
5	19.579	14.131	270, 323	Hesperidin
6	23.807	19.305	258, 378	Quercetin
7	24.243	13.965	240, 280	Cinnamic derivative
8	24.794	0.444	-	n.i.
9	25.785	0.836	267	Syringic acid
10	27.481	10.071	235, 322	Cinnamic derivative
11	28.071	5.553	252, 352, 369	Ellagic acid
12	28.517	7.221	220, 329	Rosmarinic acid

n.i.—not identified.

**Table 3:** Inhibition halos (mm) obtained in the disk diffusion test (mean ± sd) of different concentrations of *Psidium guajava* aqueous extract against the strains *Staphylococcus aureus* (resistant), *Staphylococcus aureus* (wild-type), *Staphylococcus pseudintermedius* (resistant), *Staphylococcus pseudintermedius* (wild-type), *Streptococcus* beta-hemolytic (wild-type) (n = 3).

Concentration (mg/mL)	2.3	4.6	6.8	9.1	11.4
*Staphylococcus aureus* (resistant)	14.0 ± 2.00 ^a^	15.3 ± 2.31 ^a^	16.7 ± 1.15 ^a^	17.3 ± 2.31 ^a^	18.0 ± 3.46 ^a^
*Staphylococcus aureus* (wild-type)	9.3 ± 1.15 ^a^	11.3 ± 1.15 ^a^	14.0 ± 0.00 ^b^	14.7 ± 1.15 ^b^	16.0 ± 0.00 ^b^
*Staphylococcus pseudintermedius* (resistant)	13.3 ± 1.15 ^a^	15.3 ± 1.15 ^b^	15.3 ± 1.15 ^b^	16.0 ± 0.00 ^b^	18.0 ± 0.00 ^b^
*Staphylococcus pseudintermedius* (wild-type)	13.3 ± 1.15 ^a^	15.3 ± 1.15 ^b^	15.3 ± 1.15 ^b^	16.0 ± 0.00 ^b^	18.0 ± 0.00 ^c^
*Streptococcus* beta-hemolytic (wild-type)	14.7 ± 1.15 ^a^	18.7 ± 1.53 ^a^	18.0 ± 4.0 ^a^	19.3 ± 3.06 ^a^	21.0 ± 2.0 ^a^

Equal letters do not differ significantly between the concentration (*p* > 0.05). Different letters differ significantly between the concentrations (*p <* 0.05).
